# Development and Validation of a Daily Habit Scale

**DOI:** 10.3389/fnins.2022.880023

**Published:** 2022-07-06

**Authors:** Dejan Georgiev, Rosie Christie, Mariam Torkamani, Ruifeng Song, Patricia Limousin, Marjan Jahanshahi

**Affiliations:** ^1^Department Clinical and Motor Neurosciences, Institute of Neurology, University College London, London, United Kingdom; ^2^Department of Neurology, University Medical Centre Ljubljana, Ljubljana, Slovenia; ^3^Artificial Intelligence Lab, Faculty of Computer and Information Sciences, University of Ljubljana, Ljubljana, Slovenia

**Keywords:** daily habits scale, habit formation, validation, habitual behaviour, cortico-basal ganglia-thalamo cortical loops

## Abstract

Habits are defined as automatic behaviours triggered by cues and performed without awareness. They are difficult to control and mentally efficient, which contrasts with goal-directed behaviour, which is characterised by active thought, high computational effort, and the ability to modify this behaviour in response to a changing environment and contextual demands. Habits are not only defined by the frequency with which a behaviour is performed but represent a complex construct that also includes the strength and automaticity of the habitual behaviour. We report here the development and validation of a Daily Habit Scale (DHS) to assess the frequency, automaticity, and strength of daily habits in healthy individuals. Item reduction based on factor analysis resulted in a scale with 38 items grouped into eight factors explaining 52.91% of the variance. The DHS showed very good internal consistency (*Cronbach alpha* = 0.738) and test-retest reliability (*Intraclass correlation coefficient* = 0.892, *p*<0.001) as well as convergent and divergent reliability compared to other scales measuring habits. We found a significant effect of age, gender, anxiety, and depression on the DHS. Considering certain limitations of the DHS, such as not considering the context of performance of habits, and the absence of certain items, such as transportation use, the results of this study suggest that DHS is a reliable and valid measure of daily habits that can be used by both clinicians and researchers as a measure of daily habits.

## Introduction

Habits are an essential part of our daily lives. They help us perform actions effortlessly without requiring conscious thinking, allowing us to deal with more complex problems at the same time. Habits are defined as automatic behaviours that are triggered by cues (Aarts et al., 1998; Verplanken and Orbell, 2003; Verplanken, 2006; de Wit et al., 2009; Balleine and O’Doherty, 2010; Gardner et al., 2012; Dolan and Dayan, 2013; Galla and Duckworth, 2015; Wood and Runger, 2016; Ersche et al., 2017). The automaticity of habits means that they are performed without awareness, are difficult to control, and are mentally efficient (Bargh, 1994, 1996; Verplanken and Orbell, 2003). Habitual behaviours are triggered very quickly, which is advantageous in many situations (Schneider and Chein, 2003). Because habitual behaviours are triggered by cues, they are not activated based on current goals or outcome value. A cue to a habit triggers the behaviour regardless of the consequence (Tricomi et al., 2009; Balleine and O’Doherty, 2010; Ersche et al., 2017) and is therefore difficult to resist. Habits are very stable and highly inflexible (Schneider and Chein, 2003; de Wit et al., 2009; Dolan and Dayan, 2013; Ersche et al., 2017). Habits are not only defined by the frequency of performing an action, but the construct also encompasses the strength and automaticity of the habitual behaviour (Verplanken and Orbell, 2003; Verplanken, 2006).

Unlike habitual behaviour, goal-directed behaviour is motivated and driven by its consequences. One engages in goal-directed behaviour when one wants to achieve a desired outcome and receive the rewarding consequences associated with that action (Dolan and Dayan, 2013). Goal-directed behaviour is characterised by active deliberation, high computational effort, and the ability to change that behaviour in response to a changing environment and contextual demands (Dayan, 2009). A key difference between habitual and goal-directed behaviour is that habitual behaviour is known as stimulus-response (S-R) action, meaning that a stimulus triggers a particular behaviour and will consistently lead to that behaviour (Redgrave et al., 2010; Dolan and Dayan, 2013; Robbins and Costa, 2017). Goal-directed behaviour, on the other hand, is referred to as response-outcome (R-O) action. This means engaging in a specific response to obtain a specific outcome (Redgrave et al., 2010; Dolan and Dayan, 2013).

Research on animals and humans provided evidence that habitual behaviours, as well as goal-directed behaviours are mediated by the cortico-basal ganglia-thalamo-cortical circuits (Wood and Runger, 2016; Knowlton and Patterson, 2018; Patterson and Knowlton, 2018). While the associative circuit, which also supports working memory and is connected to the prefrontal cortex, is considered to mediate goal-directed behaviours, the sensorimotor circuit underlies automatic, habitual behaviours and is connected to the somatosensory and motor cortex and supplementary motor area via the medial and posterior putamen (Wood and Runger, 2016; Knowlton and Patterson, 2018). Indeed, lesions of the dorsolateral striatum, which includes the putamen in addition to the caudate nucleus, in rodents can lead to a task braking pattern of activity during habitual runs in a maze in rodents (Smith and Graybiel, 2013). In humans, practicing movements through repetition leads to decreased activation in areas associated with goal-directed behaviour, such as the prefrontal cortex and anterior cingulate and associative parts of the basal ganglia, and to an increase in activity in the sensorimotor cortex (Wood and Runger, 2016). The activity of the sensorimotor circuit is modulated by extended brain regions. For example, dopaminergic projections from the substantia nigra to the dorsal striatum modulate habit plasticity, and in rats a lesion of the nigrostriatal pathway leads to impaired habit formation (Wood and Runger, 2016). It is therefore reasonable to expect that, for example, patients with Parkinson’s disease (PD) with nigrostriatal dopamine deficiency. will perform normally habitual behaviours such as walking in a goal-directed manner due to the impairment of their habit system (Graybiel and Rauch, 2000; Graybiel, 2008; Redgrave et al., 2010).

Habitual behaviour needs to be objectively measurable. This is important because there are many situations in which measuring habits would be particularly useful. A habit scale would also be useful for the assessment of people with PD as well as individuals with other basal ganglia disorders such as addiction or obsessive-compulsive disorder (OCD). In addition, it could be used to determine whether and how changes in daily habits are related to the development of harmful habits such as impulse control disorders (ICD) in PD, addictions, or compulsions in OCD and whether changes in daily habits can be used to assess or predict development of such atypical and harmful habits.

There has been a debate on how to measure habit strength (Hagger et al., 2015; Labrecque and Wood, 2015). Based on Gardner’s definition of habits ‘as a process by which a stimulus automatically generates an impulse toward action, based on learned stimulus-response associations’ (Gardner, 2015); different criteria for measurement of habits can be considered, including automaticity, frequency of past performance and test of cue-behaviour cognitive associations. Gardner has emphasised the automaticity component of habits, although this automaticity of a habit has a low predictive validity (Labrecque and Wood, 2015). A habit strength should predict future behaviours and strong habits should reduce the impact of consciousness on performance (Labrecque and Wood, 2015). Over the years, several scales have been developed to measure habits. The two most successful of these are the Self-Report Habit Index (SRHI) (Verplanken and Orbell, 2003) and the Creature of Habits Scale (COHS) (Ersche et al., 2017). However, both scales have a number of limitations and there remains a need for a valid and reliable scale that measures the range of habits in daily life. A crucial limitation of the SRHI is that it only measures a particular behaviour at a particular time, i.e., the administrator of the scale selects the behaviour in which he/she is interested. Even though this measure has been designed to measure the experience of automaticity and frequency of past performance, it has recently been suggested to measure automaticity only (Gardner et al., 2012; Gardner, 2015). This means that the SRHI cannot measure the frequency or strength of engagement in behavioural habits as a whole, but only a single habitual behaviour. This is problematic because some individuals may not engage in the selected behaviour, so they are excluded from the analysis or do not receive a score. Therefore, it is not possible to use the SRHI as a measure of daily habits in general. In addition, the SRHI does not consider all aspects that define habitual behaviours. For example, it does not measure whether the behaviour is performed automatically, without attention, or without regard to consequences, which are the core characteristics of habits. This suggests that the SRHI is of limited value in accurately measuring the nature, frequency, and strength of habits that people engage in on a daily basis.

The COHS also has some important limitations. The COHS consists of two subscales: routine and automatic. However, habitual behaviours are characterised by more than just these two factors. Therefore, the COHS, like the SRHI, may not accurately measure habits because some important characteristics are not captured. In addition, the COHS was designed to provide a measure of personality that considers the extent to which individuals engage in the same habitual behaviours over time, with a focus on eating behaviours. This is a clear drawback that could be overcome by a new scale that focuses on measuring multitude of habits in daily life and would provide a better understanding of a range of basic habits.

With these limitations in mind, the Daily Habit Scale (DHS) was developed to provide a more valid and reliable measure of daily habitual behaviours. The DHS aims to obtain an index of habits in daily life. As such, the DHS measures both the types of habits that individuals engage in and the strength of those habits, while measuring the defining criteria of a habit (Verplanken and Orbell, 2003; Verplanken, 2006; Gardner et al., 2012; Gardner, 2015): (i) Frequency of engagement, (ii) Automaticity of performance, (iii) Difficulty in resisting doing it, and (iv) Unconcern about consequences. In this paper, we report on the development and validation of the DHS.

## Materials and Methods

Ethical approval for the study was obtained from the local ethics committee. Participants gave informed consent. Participants were recruited via the online participant pools Prolific and Call for Participants. Data were collected using the Qualtrics online system and subsequently stored in an electronic database.

### Participants

Six hundred and sixty-four participants took part in the present study. The response rate calculated as the percent of people who had completed the survey relative to those who had clicked on/accessed the survey was 78.03%. Seventy-nine participants (11.9%) were excluded from the analysis because they reported having suffered a head injury, abusing drugs or alcohol, suffering from a neurological or mental illness, or taking medication that affects the nervous system. This resulted in 585 participants (female: 281, male: 301, prefer not to say: 3; right-handed: 523, left-handed: 62; mean age: 35.03 ± 17.42, range: 18-84) who were included in the analysis. All participants are healthy individuals.

One hundred and seventeen participants completed the DHS retest. Using the above exclusion criteria, 18 participants (15.38%) were removed from the retest analysis. This left a sample of 99 individuals (female: 75, male: 23, prefer not to say: 1; right-handed: 90, left-handed: 9; mean age: 42.93 ± 19.47, range: 18-81). There were no significant differences in age, *t* = 2.21, *p* = 0.065, but there was a significant difference in gender between the test and retest groups, *X*^2^ (1) = 31.89, *p* < 0.001.

### Procedure

A link to the survey was posted to the participants. An information sheet was presented first, followed by an informed consent form. Before the survey began, participants were required to provide a self-generated unique identification code. This was used to match participants to the second repeat survey to assess the test-retest reliability of the DHS. Participants then completed a number of demographic questions, such as age, gender, marital status, handedness, employment status, occupation, information on head injuries, drug/alcohol abuse, or diagnoses of neurological conditions, and finally they provided information on medications they were taking.

Participants then completed each of the four questionnaires, in order, DHS, Questionnaire for Impulsive Disorders in Parkinson’s disease (QUIP) (Weintraub et al., 2009), Hospital Anxiety and Depression Scale (HADS) (Zigmond and Snaith, 1983) and COHS (Ersche et al., 2017). At the beginning of each questionnaire, a brief “instruction” described how to complete each questionnaire.

At the end of this survey, participants could decide whether they wanted to participate in a follow-up evaluation to assess the test-retest reliability of the DHS. If they wished to do so, they provided their email address so they could be contacted with a link to the second completion of the DHS. Two weeks after completing the DHS for the first time, participants were emailed a link to complete the DHS again, also through Qualtrics. An information sheet was provided, and participants gave their consent. Participants entered their unique identification code that they had included in the first survey. Then participants completed the DHS for the second time.

### Other Questionnaires Completed

#### Questionnaire for Impulsive Disorders in Parkinson’s Disease

The QUIP (Weintraub et al., 2009) consists of three sections. However, the third section of the QUIP was removed in the present study because this section refers to PD medications, which would not be relevant for healthy participants. The first section captures ICDs with five items related to four impulsive behaviours: Gambling, Sex, Buying, and Eating. The second section assesses other behaviours: Punding: an intense interest in meaningless activities; Hobbyism: a form of punding characterised by an intense interest in a particular activity or hobby; and Walkabout: excessive aimless wandering (Weintraub et al., 2009). The QUIP provides a score for each of the seven behaviours assessed. A positive score for compulsive gambling is assigned if two of the five gambling items were affirmed. Compulsive sexual behaviour was assigned a positive score if one of the five sexual behaviour items was affirmed. Compulsive buying scored as a positive outcome if any of the five buying behaviour items were affirmed. Finally, compulsive eating was scored as a positive outcome if two of the five eating attributes were affirmed. Hobbyism, Punding, and Walkabout received a positive outcome when each item was affirmed.

#### Creature of Habit Scale

The COHS (Ersche et al., 2017) includes 27 items. For example, item 1. “I like to park my car or bike always in the same place,” or item 4. “I tend to go to bed at roughly the same time every night.” These are scored on a 5-point scale ranging from “strongly agree” = 5 to “strongly disagree” = 1, with some items scored inversely. The COHS is divided into two subscales: *Routine* and *Automaticity*. Scores on the *Routine* subscale can range from 16 to 80. Scores on the *Automaticity* subscale can range from 11 to 55. Higher scores reflect a stronger expression of habitual behaviours.

#### Hospital Anxiety and Depression Scale

Anxiety and depression were measured using the HADS (Zigmond and Snaith, 1983), which consists of 14 items: seven assessing anxiety and the other seven assessing depression. Each item is scored on a scale of 0–3. Higher scores on both the anxiety and depression scales represent more severe symptoms. On both the anxiety and depression scales, scores of 0–7 are within the normal range. For both anxiety and depression, a score of 8–10 is considered borderline abnormal. For both anxiety and depression, a score of 11 or above is considered abnormal. This cut-off was used to divide the group into low (< 11) and high (≥ 11) anxiety and depression subgroups.

### Development and Validation of the Daily Habit Scale

A three-step approach was used to develop and validate the DHS: 1. Item generation; 2. Item reduction; 3. Scale validation and reliability testing.

### Item Generation

Items for the DHS were selected by reviewing the literature on daily habits. Through expert group discussion other items were generated as they were thought to meet the criteria used to define habits. This allowed a preliminary scale to be generated, which was then discussed by a panel of experts in neuropsychology (three experts) and neurology (three experts). As a result, a scale with 40 items was developed - the Daily Habit Scale (DHS). The DHS assesses each participant’s daily habits by collecting information about daily habits such as drinking water, shopping, and exercising, checking emails, resting etc. Except for the last two questions, each item was a closed-ended question that began with two options: – For example, item 1. “Smoking is something that…”, or item 13. “Waking up early is something that…” *I do not do at all* (go to the next question) or *I do* (answer questions bellow) on a four point scale: *I do several times a day/daily/weekly/monthly* (please circle the choice), which allowed presence/absence of particular habitual behaviour and quantifying its frequency. The automaticity of each habitual behaviour was then rated by ticking all the appropriate/applicable of four options: *I do automatically/without thinking, I start doing before a realize I am doing it, I would find hard not to, I would continue doing regardless of any consequence.* The two open-ended questions had the same structure. The only difference from the closed-ended questions was that participants were asked to indicate a specific habit that was not listed on the scale. For each item, frequency was scored from 0 to 5 (0 = I do not do at all, 1 = I do, 2 = I do monthly, 3 = I do weekly, 4 = I do daily, 5 = I do several times a day). Each of the four applicable intensity options is given a score of 1 which are added together. From the frequency (F) and automaticity (A). Then, the strength of the habit (S) was calculated as a mean of both measures [S = (F+A)/2]. A total strength score for the DHS is calculated by adding the strength for all applicable items. The DHS score can range from 0 to 180 (or 0 to 171 excluding the last two open-ended questions). A higher score indicates a higher frequency and automaticity, and hence strength of daily habitual behaviours.

### Item Reduction

Open-ended questions 39 and 40 were excluded because the responses to these questions varied widely among participants and could not be readily categorised. Correlational analysis using Spearman correlation coefficient (ρ) was performed to exclude possible items with low correlation with the other items. All items correlated well with each other. An item having significant correlations with at least a third of the other items was used as a criterion for retaining it. Thus, this did not result, in exclusion of any more items and all 38 items were retained for further analysis. The percentage of responses to the 38 closed-ended questions in the test and retest datasets is shown in Supplementary Tables 1A,B, respectively.

### Scale Validation and Reliability Testing

Construct validity was assessed using exploratory factor analysis to determine the key components of the 38-item questionnaire. Orthogonal varimax rotation with Kaiser normalisation and an eigenvalue cut-off point > 1 was used to extract the underlying factors. Internal consistency was assessed using Cronbach’s coefficient alpha (α). Test-retest reliability was assessed using the intraclass correlation coefficient (*ICC*). Concurrent validity was assessed by measuring the Spearman correlation coefficient (ρ) of the underlying subscales of the DHS with the subscales of QUIP, HADS, and COHS. Unpaired *t*-test was used to compare the values of subgroups based on age, and anxiety. The *X*^2^ test was used to compare gender between the test and retest group. Alpha level of 0.05 was considered statistically significant. False discovery rate (Benjamini and Hochberg, 1995) to correct for multiple comparisons was used. SPSS for Mac v.26 was used to analyse the data.

## Results

### Construct Validity

Factor analysis revealed eight factors (Table 1) with eigenvalues greater than 1.0, which explained a total of 52.91% of the variance. Expert opinion was sought throughout the process to ensure that the grouping of items into factors made sense and for labelling each factor. All factors were well correlated with each other (please see Supplementary Table 2A for the test and Supplementary Table 2B for the retest datasets).

**TABLE 1:** The eight factors of the Daily Habit Scale, their descriptive labels, eigenvalues, and percent of variance accounted for. For each factor, the items loaded on that factor and their respective factor loadings are also shown.

Factor number	Factor name	Item number	Item description	Factor loading	Eigenvalue	Percent variance
F1	Hygiene and self-care activities	20	Washing my hands	0.744	7.121	11.688
		19	Brushing my hair	0.742		
		22	Putting on makeup/parfum	0.727		
		18	Brushing my teeth	0.723		
		23	Having bath/shower	0.652		
		3	Drinking water	0.443		
		21	Shaving	0.401		
F2	Leisure activities	38	Playing games	0.655	3.489	9.595
		5	Chewing gum	0.561		
		11	Having a rest	0.561		
		30	Surfing internet	0.481		
		35	Listening to music	0.463		
		27	Watching TV	0.401		
F3	Household activities	16	Carrying out household chores	0.809	2.603	6.839
		17	Cleaning/tidying	0.728		
		24	Emptying bowels	0.666		
		15	Gesturing	0.408		
F4	Common daily activities	9	Taking my medication prescribed	0.671	1.902	6.307
		10	Taking pain killers	0.561		
		4	Drinking tea/coffee	0.517		
		34	Reading newspaper	0.507		
		25	Shopping	0.409		
F5	Unhealthy habits	7	Eating chocolate/sweets	0.656	1.542	5.653
		2	Having alcoholic drink	0.653		
		8	Eating savoury snacks/crisps	0.651		
		26	Spending money	0.616		
		14	Having sex	0.584		
		6	Eating fast food	0.466		
		1	Smoking	0.401		
F6	Sport-related activities	37	Participating in sports	0.719	1.271	4.834
		36	Exercising	0.701		
		32	Socialising	0.408		
		29	Calling particular people	0.401		
F7	Technology and internet use	31	Checking emails	0.532	1.138	4.178
		28	Using my mobile phone	0.407		
		33	Internet social networking	0.401		
F8	Sleep-related activities	12	Sleeping late	0.691	1.038	3.801
		13	Waking up early	–0.791		

### Internal Consistency

The overall Cronbach’s alpha was α = 0.738, indicating that the DHS had very high internal consistency. Each of the eight factors of the DHS had high internal consistency (Table 2). This indicates that each factor is important in maintaining the high level of overall internal consistency.

**TABLE 2:** Factor total correlations and Cronbach’s alpha if factor deleted for each the eight factors of the Daily Habit Scale.

Factor	Item total correlation	Cronbach’s alpha if factor deleted
Hygiene and self-care activities	0.522	0.692
Leisure activities	0.388	0.723
Household activities	0.503	0.697
Common daily activities	0.319	0.735
Unhealthy habits	0.492	0.699
Sport-related activities	0.416	0.715
Technology and internet use	0.567	0.695
Sleep-related activities	0.359	0.731

### Test-Retest Reliability

Test-retest reliability was analysed by correlating completions of the DHS at Time 1 with those at Time 2 (two weeks later) by 99 participants whose demographic characteristics were broadly representative of the entire sample. This yielded a high and significant *ICC* = 0.892, with a 95% confidence interval from 0.839 to 0.928, *F*(98) = 9.290, *p* < 0.001. Because of the significantly different gender structure of the test and retest group, we also redid the test-retest reliability analysis separately by gender. The overall test-retest reliability was significant in both females, *ICC* = 0.890, with a 95% confidence interval from 0.827 to 0.930, *F*(75) = 9.117, *p* < 0.001, and males, *ICC* = 0.897, with a 95% confidence interval from 0.747 to 0.958, *F*(20) = 9.741, *p* < 0.001. The high correlation indicates that the test-retest reliability of the DHS is good. Moreover, the test-retest reliability of each of the nine factors was high and all had high and significant correlations regardless of the gender of the subjects (Table 3).

**TABLE 3:** Test-retest reliability of the eight factors of the Daily Habit Scale expressed as interclass correlation coefficients and the associated *p*-values in all subjects (*n* = 99) and separately in females (*n* = 75) and males (*n* = 23).

Factor	All	Females	Males
Hygiene and self-care activities	0.658, *p* < 0.001	0.618, *p* < 0.001	0.786, *p* = 0.002
Leisure activities	0.896, *p* < 0.001	0.884, *p* < 0.001	0.923, *p* < 0.001
Household activities	0.744, *p* < 0.001	0.678, *p* < 0.001	0.871, *p* < 0.001
Common daily activities	0.875, *p* < 0.001	0.876, *p* < 0.001	0.865, *p* < 0.001
Unhealthy habits	0.874, *p* < 0.001	0.877, *p* < 0.001	0.871, *p <* 0.001
Sport-related activities	0.839, *p* < 0.001	0.842, *p* < 0.001	0.839, *p* < 0.001
Technology and internet use	0.874, *p* < 0.001	0.837, *p* < 0.001	0.816, *p <* 0.001
Sleep-related activities	0.694, *p* < 0.001	0.711, *p* < 0.001	0.614, *p* = 0.021

*One subject preferred not to state the gender and was not included in the analysis.*

### Convergent and Divergent Validity

Although the correlations were not very large in magnitude, many of them were significant. Of greatest interest are the significant positive associations between the DHS and the other two habit measures, the COHS (for both, Routine, ρ = 0.114, *p* = 0.015 and Automaticity, ρ = 0.166, *p* < 0.001 subscales) and the QUIP (for Gambling, ρ = –0.090, *p* = 0.030, and Eating, ρ = 0.135, *p* < 0.001), but not for Sex, Buying, Hobbysm, Punding, and Walkabout, all *p* > 0.195), confirming the convergent validity of the DHS. There was also a positive association between the DHS and the HADS anxiety subscale, ρ = 0.127, *p* = 0.002. More specifically, there was a significant correlation between anxiety and five of the DHS subscales: *Hygiene and self-care activities* subscale ρ = 0.097, *p* = 0.019, *Leisure activities* subscale ρ = 0.177, *p* < 0.001, *Unhealthy habits* subscale ρ = 0.192, *p* < 0.001, *Sport-related activities* subscale ρ = –0.112, *p* = 0.007, *Technology and internet* use subscale ρ = 0.180, *p <* 0.001. The correlation between the DHS and the depression HADS subscale was also significant, ρ = –0.130, *p* = 0.002. This was due to the significant correlation between depression and *Hygiene and self-care activities* subscale ρ = –0.095, *p* = 0.021, *Leisure activities* subscale ρ = 0.085, *p* = 0.039, *Household activities* subscale ρ = –0.141, *p* = 0.001, *Common daily activities* subscale ρ = –0.160 *p* < 0.001, and *Sport-related activities* subscale ρ = –0.306, *p* < 0.001. The positive association of the DHS with the HADS anxiety and the negative association with the HADS depression score confirm the divergent validity of the DHS.

### The Effect of Anxiety on the Daily Habit Scale

**FIGURE 1:**
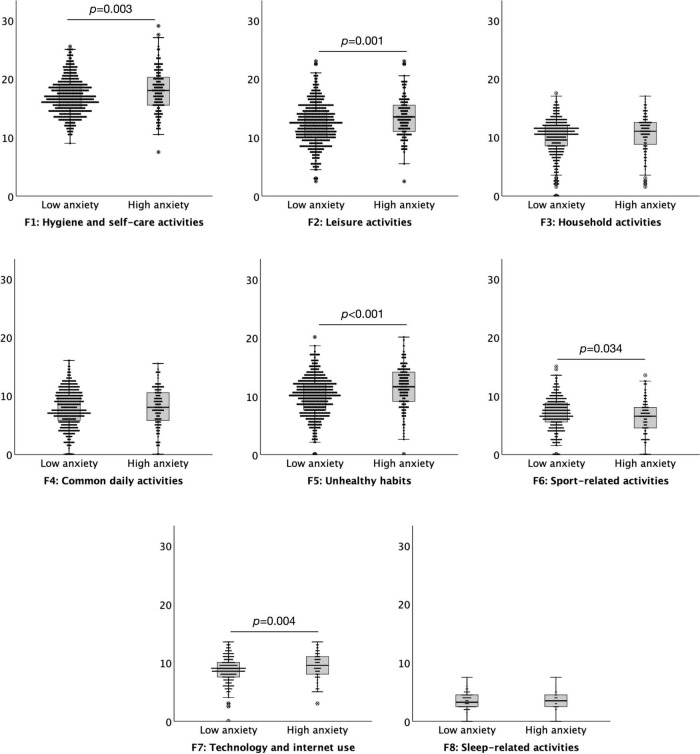
Boxplots (95% confidence interval, 25%–75% percentiles, median) of the factors of the Daily Habit Scale Factors (y-axis) in subjects with low (< 11) (*n* = 460) and high (≥ 11) (*n* = 125) anxiety levels (x-axis), as assessed by the anxiety subscale of the Hospital Anxiety and Depression Scale.

A commonly used cut off score of 11 on the HADS anxiety subscale was used to define high (abnormal ≥ 11) versus low (normal < 11) levels of anxiety suggestive of “caseness.” There were 125 (21.37%) participants in the high anxiety group and 460 (78.63%) in the low anxiety group. The results indicated that individuals with higher levels of anxiety had a significantly increased level of daily habitual behaviours, *M* = 80.55, *SD* = 15.61, than those with low levels of anxiety, *M* = 76.09, *SD* = 13.24, *t* = 3.209, *p* = 0.001. Higher levels of anxiety resulted in significantly higher levels of engagement in habits compared to low anxiety on five subscales of the DHS: *Hygiene and self-care activities t* = 3.020, *p* = 0.003, *Leisure activities t* = 3.342, *p* = 0.001, *Unhealthy habits t* = 4.149, *p* < 0.001, *Technology and internet use t* = 2.864, *p* = 0.004, and significantly lower level of engagement in *Sport-related activities t* = –2.128, *p* = 0.034. There were no significant differences between subjects of low and high anxiety levels for *Household activities, Common daily activities*, or *Sleep-related activities*, all *p* > 0.078 (Figure 1).

### The Effect of Depression on the Hospital Anxiety and Depression Scale

**FIGURE 2:**
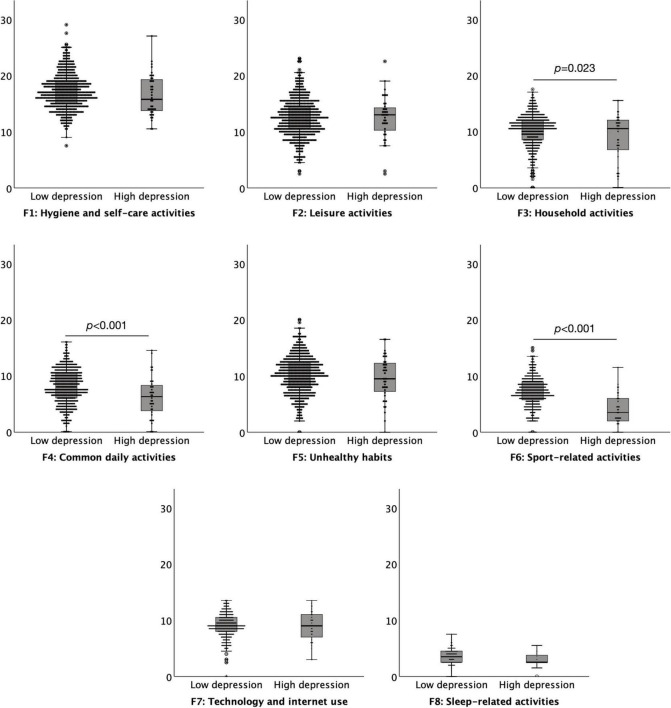
Boxplots (95% confidence interval, 25%–75% percentiles, median) of the factors of the Daily Habit Scale Factors (y-axis) in subjects with low (< 11) (*n* = 544) and high (≥ 11) (*n* = 41) depression levels (x-axis), as assessed by the depression subscale of the Hospital Anxiety and Depression Scale.

A commonly used cut off score of 11 on the HADS depression subscale was used to define high (abnormal ≥ 11) versus low (normal < 11) levels of depression suggestive of ‘caseness’. There were 41 (7.00%) participants in the high depression group and 544 (93.00%) in the low depression group. The results indicated that individuals with lower levels of depression had a significantly increased level of daily habitual behaviours, *M* = 77.63, *SD* = 13.48, than those with high levels of anxiety, *M* = 69.35, *SD* = 16.89, *t* = 3.717, *p* < 0.001. Lower levels of depression resulted in significantly higher levels of engagement in habits compared to high depression on three subscales of the DHS: *Household activities t* = –2.280, *p* = 0.023, *Common daily activities t = –*3.705, *p* < 0.001, and *Sport-related activities t = –*7.318, *p* < 0.001. There were no significant differences between subjects of low and high anxiety levels for *Hygiene and self-care activities*, *Leisure activities*, *Unhealthy habits*, *Technology and internet use*, or *Sleep-related activities*, all *p* > 0.079 (Figure 2).

### The Effect of Age and Gender on the Daily Habit Scale

Age had a significant negative correlation with strength of daily habits ρ = –0.138, *p* = 0.041. In fact, age had a significant negative correlation with *Hygiene and self-care activities* subscale ρ = –0.146, *p* < 0.001, *Leisure activities* subscale ρ = –0.357, *p* < 0.001, *Unhealthy habits* subscale ρ = –0.206, *p* < 0.001, *Technology and internet use* subscale ρ = –0.183, *p* < 0.001 and *Sleep-related activities* subscale ρ = –0.196, *p* < 0.001. In contrast, age had a significant positive correlation with the *Household activities* subscale ρ = 0.149, *p* < 0.001, and *Common daily activities* subscale ρ = 0.483, *p* < 0.001. The correlation between age and *Sport-related activities* subscale was not significant ρ = 0.035, *p* = 0.393. Similarly, in the retest sample, age correlated negatively with the daily habit scale ρ = –0.222, *p* = 0.020, and it subscales *Hygiene and self-care activities ρ* = –0.351, *p* < 0.001, *Leisure activities ρ* = –0.249, *p* = 0.013, *Unhealthy habits ρ* = –0.269, *p* = 0.007, *Technology an internet use ρ* = –0.208, *p* = 0.039 and *Sleep-related activities ρ* = –0.306, *p* = 0.002. There was also a positive correlation of age with the *Common daily activities* subscale ρ = 0.352, *p* < 0.001. The correlation of age with the *Household cleaning* subscale was significance in the retest sample ρ = 0.003 *p* = 0.935.

**FIGURE 3:**
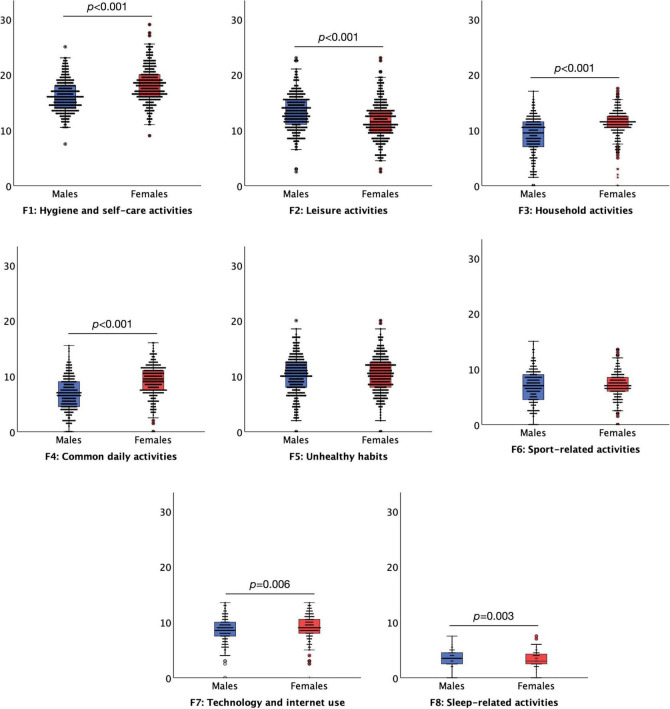
Boxplots (95% confidence interval, 25%–75% percentiles, median) of the factors of the Daily Habit Scale Factors (y-axis) in female (red boxplots) (*n* = 281) and male (blue boxplots) (*n* = 301) (x-axis) subjects. Three subjects preferred not to say their gender.

Regarding the effect of gender, the results suggested that females overall (*n* = 281, 48.00 of the whole sample) engaged more in the daily habitual behaviours, *M* = 79.91, *SD* = 13.27, than males (*n* = 301, 51.45% of the whole sample), *M* = 74.21, *SD* = 13.70, *t* = 5.078, *p* < 0.001. Females had significantly higher levels of daily habitual behaviours on the *Hygiene and self-care activities t* = 8.824, *p* < 0.001, *Household activates t* = 8.349, *p* < 0.001, *Common daily activates t* = 9.754, *p* < 0.001, *Technology an internet use t* = 2.734, *p* = 0.006 subscales than males. By contrast, males had significantly higher levels of daily habitual behaviour on the *Leisure activities t* = 5.143, *p* < 0.001 and *Sleep-related activities, t* = 3.082, *p* = 0.003 subscales compared to females. Gender did not significantly affect *Unhealthy habits* and *Sport-related activities*, all *p* > 0.172 (Figure 3). In the retest sample, females (*n* = 75, 75.75% of the whole sample) and males (*n* = 23, 23.23%) did not differ in the overall daily habitual daily behaviours, *M* = 78.31, *SD* = 11.21 and *M* = 87.56, *SD* = 11.21, respectively, *t* = 0.265, *p* = 0.787. Also, in the retest sample the differences between females and males for the levels of habitual daily behaviours for the subscales did not reach significance (all *p* > 0.069).

## Discussion

The aim of this study was to develop and validate the DHS. We developed a 38-item scale. The results showed that the DHS is a valid and reliable scale for measuring daily life habits. Factors analysis of the 38 items, showed its construct validity with the main variance captured by eight key factors, with high internal consistency and test-retest reliability.

Two scales relating to “normal” and pathological habits, namely the COHS and the QUIP, were used to assess the convergent validity of the DHS. There was a significant positive correlation between the two subscales of the COHS, *routine* and *automaticity*, and the DHS. This was as expected since both scales measure habitual behaviours. There were also significant correlations between two of the subscales of the QUIP, particularly *impulsive eating*, and *gambling*, and the DHS. The QUIP also measures habitual behaviours, albeit pathological habits such as gambling, and impulsive eating; thus, these significant correlations were also expected and predicted. However, it is worth noting that the correlations between the DHS and the COHS and QUIP were low, although significant. This may be due in part to the fact that each measure reflects specific aspects of habits, e.g., eating habits for COHS and abnormal and pathological habits for QUIP.

As expected, there was a significant correlation between the HADS anxiety subscale and the DHS. Namely, previous evidence suggests that anxious individuals engage in more habitual behaviours than people without anxiety (Alvares et al., 2014; Ersche et al., 2017). However, these previous findings were based on the use of different habit scales and some focused on social anxiety rather than general anxiety. Nonetheless, the present study confirms that higher levels of general anxiety lead to higher engagement in everyday daily habits. This relationship could also be explained by the results of animal studies, which suggest that anxiety leads to frontal lobe degeneration, which in turn results in disruption of the formation and maintenance of goal-directed behaviours (Dias-Ferreira et al., 2009). With lower engagement in goal-directed behaviours, habitual behaviours must take over, which would lead people with anxiety to engage in more habitual behaviours than people without anxiety. This result also confirms the convergent validity of the DHS. Moreover, the correlation of the DHS with the HADS depression subscale was negative and significant, as expected, suggesting that individuals with lower levels of depression have a significantly higher level of daily habit behaviours. This can be explained by the fact that lack of interest and motivation, characteristic symptoms of depression, reduce engagement in goal-directed behaviours and thus impair engagement in habitual behaviours. However, the evidence to date for a link between depression and habits is rather indirect and contradictory. There is some evidence for differences in striatal dopamine in depressed patients (Willner, 1983; Bowden et al., 1997; Santiago et al., 2014). As striatal dopamine is likely to be a key factor in habitual behaviours, this could have implications for daily habits. However, evidence also exists that striatal dopamine activity does not differ in depressed patients (Hirvonen et al., 2008). Indeed, in our study lower level of depression resulted in significantly higher engagements in some (*Household activities*, *Common daily activities*, and *Sport-related activities*), but not all DHS subscales The DHS and the HADS depression subscale measure different constructs and therefore it is important to note that they were not positively correlated with each other. This confirmed the divergent validity of the DHS.

There was a significant negative correlation between age and the total DHS score. More specifically, age negatively correlated with certain subscales, *Hygiene and self-care activities Leisure activities, Unhealthy habits*, *Technology and internet use* and *Sleep-related activities*. Younger people would be expected to engage more in technology and internet use as they are typically more technologically knowledgeable than older populations (Olson et al., 2011). It also makes sense that younger people engaged in more leisure activities as this subscale involved items such as playing games which are likely to appeal more to the younger population and are perhaps more available to them. The majority of virtual games are targeted at younger people, and they are likely to have access to them on different devices and game consoles. Younger people also spend more time in self-care and hygiene activities. Unhealthy habits such as eating chocolate, eating savoury snacks, and fast food, as well as smoking and spending money are more common among younger people. This subscale also included an item on sexual intercourse, which is not an unhealthy habit in itself but, if not protected, for example can be risky and lead to health complications and loaded within this subscale. Research has shown that younger people engage in more sexual intercourse than older people (Pearlman and Kobashi, 1972; Pfeiffer and Davis, 1972). Finally, the reason for younger people engaging in more sleep-related habits may be because the sleep cycle changes with age (Feinberg, 1974; Cajochen et al., 2006) and so sleep duration is shorter and is more interrupted in older individuals and which could reduce the habits related to sleep. On the other hand, the results suggest that older people engage in more *Household cleaning* and *Common daily activities*, including taking medication. The latter would fit with expectations and the available data, as older people tend to have more medical issues and are therefore on more prescription medication than younger people and may also have to take more pain killers. Older people engaging in more household cleaning than younger people could be due to several reasons. One main reason could be because a larger proportion of younger people may still live with their parents who are likely to be in charge of the household activities.

We found that females engaged more in daily habit behaviours than males. This is in concordance to previous research that suggested that the neural correlates behind habits, i.e., levels of striatal dopamine activity, were stronger in females than males (Mozley et al., 2001; Laakso et al., 2002; Haaxma et al., 2007). More specifically, females were more engaged in *Hygiene and self-care activities*, *Household activities, Common daily activities and Technology and internet use* than males. Household cleaning is stereotypically perceived as more of a female role, and the higher score of females on this scale seems to reflect higher engagement in this. Similarly, females tend to spend more time on self-care activities, such as make-up and brushing hair, than males. Even when it comes to common daily activities, like shopping and taking medication, the odds are in favour of females, the explanation for which seems reasonable, because females use contraceptive pill or hormone replacement therapy and are more fond of shopping than males (Merrell, 2003). At a first glance, it looks like technology and internet use may be more common among males. However, this subscale included the use of mobile phone, checking e-mails and internet social networking, all of which are likely to be more prevalent among females. This could be due to gender differences in sociability, as females tend to be more sociable and enter relationships more easily than males. For example, females perceive same-sex social interactions more rewarding than males, and activation of oxytocin receptors in the ventral tegmental area is critical for social reward in both females and males (Borland et al., 2019). On the other hand, males engaged in more *Leisure activities* and *Sleep-related activities.* Males play more games than females (López-Fernández et al., 2021), and they are more prone to sedentary type of daily activities (Segrin, 2000), including watching television than females. In addition, gender differences in sleep patterns with females having longer sleep times, shorter sleep-onset latency and higher sleep efficiency (Krishnan and Collop, 2006) could explain the gender differences in *Sleep-related activities* (encompassing the items *Sleeping late* and *Waking up early*) in favour of men.

The DHS can be potentially used in different populations. Habitual behaviours are strongly affected in PD (Knowlton et al., 1996; Witt et al., 2002; Wilkinson et al., 2008, 2009). The DHS will be useful for assessing people with PD. It has been suggested that people with PD perform normally habitual behaviours such as walking in a goal-directed manner due to the overuse and impairment of their habitual system (Redgrave et al., 2010; Hernandez et al., 2019). Thus, the DHS can be used in the prodromal phase of PD or in individuals with rapid eye movement sleep disorder who then develop PD (Postuma et al., 2012) to determine gradual overuse and decline of the habitual system over time. Researchers could use the DHS to understand whether and. how habits change during the progression of PD. This could provide crucial clues to how PD evolves and changes the balance between goal-directed and habitual behaviours. This is an excellent starting point to better understand the mechanisms behind PD and its links with the pattern of dopamine loss that begins in the sensorimotor dorsal striatum and is associated with habits (Wood and Runger, 2016; Hernandez et al., 2019). In such longitudinal studies, the DHS can also be used as a validated questionnaire to understand how engagement in daily habits changes with initial onset and further development and progression of symptoms of PD. Harmful habits in the form of ICDs are relatively common in PD treated with dopamine medications, affecting 15-20% of these patients (Weintraub et al., 2015). ICDs are also common in the general population. There is evidence of a lifetime prevalence of 9.2% (de Graaf et al., 2012). Therefore, DHS can be used to quantify daily habits and determine if any changes in these, may relate to development of ICDs.

The DHS can also be used as a questionnaire to assess and understand other disorders in which habits may be affected, such as addictions (Belin et al., 2013), Gilles da la Tourette syndrome (GTS) (Martino and Hedderly, 2019), or OCD (Gillan and Robbins, 2014; Hadjas et al., 2019). The scale could be used to assess change in symptoms such as tics and compulsions which represent habitual behaviours and potentially provide new information about the development of such harmful habits and the course of these disorders. DHS can be a useful tool in changing certain maladaptive behaviours. For example, a previous study suggested the need for a habit scale to implement behavioural strategies for injury prevention (Nilsen et al., 2008). To develop a behavioural strategy, it is important to understand the nature and strength of current habits. Therefore, in such situations, the DHS could be a very useful tool to gain a better understanding of the mechanisms behind the formation and maintenance of habits in health and disease.

The DHS could also be used more generally to develop a better understanding of habit formation, maintenance, and strength in the general population. Therefore, in addition to being used in a clinical setting, the DHS can be very useful for future research.

Analysis of the influence of key factors provides some useful insights into the study of habits. The results suggest that age and anxiety have the greatest influence on habitual behaviours, while gender and depression have no influence. These findings could be useful when using the DHS with clinical populations such as patients with PD, OCD, addictions, or GTS, as clinicians and researchers will have a better idea of what factors do not influence habits and what factors may need to be controlled when using the DHS. It is also useful to know that of the variables tested, age is the greatest predictor of daily habits. Future research could also examine other factors that might influence daily habitual behaviour. These could include variables such as personality, presence/absence of ICDs, and other neurological or neurological disorders. This would help to understand the impact of these factors on daily habits. It would also be useful to investigate other factors to improve the predictive model for DHS. The inclusion of other psychological, social, and environmental factors would likely increase the amount of variance in DHS that could be explained. This in turn could lead to a better understanding of the nature and determinants of natural daily habits in health and disease.

This study is subject to certain limitations. For example, some important habitual activities, such as use of transportation, identified in previous work (Wood et al., 2002) have not been included. Nevertheless, we believe that the fact that the DHS includes 38 items across nine different factors makes the scale sufficiently broad to reflect a wide range of daily habitual behaviours. In addition, certain items that one would expect to be grouped into a single factor, such as *Internet social networking*, *Socialising*, and *Calling particular people* were distributed across different factors, namely *Technology and internet use* and *Sport-related activities*. It is easy to understand that *Internet social networking* was loaded on the *Technology and internet use* factor, since social networking nowadays depends heavily on the use of cell phones and computers. On the other hand, *Socialising* and *Calling particular people*, together with *Participating in sports* and *Exercising* grouped together with to the factor *Sports-related activities*, which can be explained by the fact that participation in sports activities usually requires contact with certain people who are interested in the same sport. There were also inconsistencies in other item-factor loadings, such as *Drinking water* in *Hygiene and self-care activities* that could have been expected to be loaded to *Common daily activities*, or *Emptying bowls* in *Household activities* instead of *Common daily activities* or *Hygiene and Self-related activities*. In addition, *Having sex* loaded to *Unhealthy habits*. As explained earlier, *Having sex* itself is not an unhealthy habit, but it could lead to health problems if it is not practiced properly, such as when it is done unprotected. Nevertheless, the overall structure of the DHS is congruent, and explanations can be found even for items that are counterintuitive and load to unexpected factors. The DHS does not account for performance context, which is a central component of habit formation and habit activation, because repeated performance in different contexts requires decision making and thus do not reflect habit. Although performance context may be theoretically important, particularly for habit formation, we did not consider it relevant for measuring the strength of daily habits. Performance context cannot be easily or meaningfully operationalized within the context of a scale.

In conclusion, the present study developed and validated the DHS. The results show that the 38 item DHS consists of eight factors. The DHS was found to have high construct validity, internal consistency, test-retest reliability, convergent validity, and divergent validity. Therefore, considering certain limitations of the DHS, such as not considering the context of performance of habits, and the absence of certain items, e.g., transportation use, the DHS can be used by both clinicians and researchers as a measure of everyday daily habits.

## Data Availability Statement

The raw data supporting the conclusions of this article will be made available by the authors, without undue reservation.

## Ethics Statement

The studies involving human participants were reviewed and approved by University College London. The patients/participants provided their written informed consent to participate in this study.

## Author Contributions

DG, MJ, RC, and PL had substantial contributions to the conception or design of the work, the acquisition, analysis or interpretation of data for the work, provided approval for publication of the content, agreed to be accountable for all aspects of the work in ensuring that questions related to the accuracy, or integrity of any part of the work are appropriately investigated and resolved. MT and RS contributed substantially to the conception of the work and interpretation of the data for the work and agreed to be accountable for all aspects of the work in ensuring that questions related to the accuracy, or integrity of any part of the work are appropriately investigated and resolved. DG and MJ drafted the work or revisited it critically for important intellectual content. All authors contributed to the article and approved the submitted version.

## Conflict of Interest

The authors declare that the research was conducted in the absence of any commercial or financial relationships that could be construed as a potential conflict of interest.

## Publisher’s Note

All claims expressed in this article are solely those of the authors and do not necessarily represent those of their affiliated organizations, or those of the publisher, the editors and the reviewers. Any product that may be evaluated in this article, or claim that may be made by its manufacturer, is not guaranteed or endorsed by the publisher.
